# *Lactobacillus fermentum* ME-3 – an antimicrobial and antioxidative probiotic

**DOI:** 10.1080/08910600902815561

**Published:** 2009-03-16

**Authors:** Marika Mikelsaar, Mihkel Zilmer

**Affiliations:** Department of Microbiology and Department of Biochemistry, University of Tartu, Tartu, Estonia

**Keywords:** animal experiments, antimicrobial activity, antioxidative activity, atherosclerosis, atopic dermatitis, blood lipids, cerebral stroke, enteric infections, food supplements, functional food, glutathione, human clinical trials, intestinal microbiota, inflammation, isoprostanes, lactobacilli, Lactobacillus fermentum ME-3, oxidative stress, oxidized LDL, post-prandial lipidaemia, probiotics, probiotic patents, typhoid nodules

## Abstract

The paper lays out the short scientific history and characteristics of the new probiotic *Lactobacillus fermentum* strain ME-3 DSM-14241, elaborated according to the regulations of WHO/FAO (2002). *L. fermentum* ME-3 is a unique strain of *Lactobacillus* species, having at the same time the antimicrobial and physiologically effective antioxidative properties and expressing health-promoting characteristics if consumed. Tartu University has patented this strain in Estonia (priority June 2001, patent in 2006), Russia (patent in 2006) and the USA (patent in 2007). The paper describes the process of the identification and molecular typing of this probiotic strain of human origin, its deposition in an international culture collection, and its safety assessment by laboratory tests and testing on experimental animals and volunteers. It has been established that *L. fermentum* strain ME-3 has double functional properties: antimicrobial activity against intestinal pathogens and high total antioxidative activity (TAA) and total antioxidative status (TAS) of intact cells and lysates, and it is characterized by a complete glutathione system: synthesis, uptake and redox turnover. The functional efficacy of the antimicrobial and antioxidative probiotic has been proven by the eradication of salmonellas and the reduction of liver and spleen granulomas in *Salmonella* Typhimurium-infected mice treated with the combination of ofloxacin and *L. fermentum* strain ME-3. Using capsules or foodstuffs enriched with *L. fermentum* ME-3, different clinical study designs (including double-blind, placebo-controlled, crossover studies) and different subjects (healthy volunteers, allergic patients and those recovering from a stroke), it has been shown that this probiotic increased the antioxidative activity of sera and improved the composition of the low-density lipid particles (LDL) and post-prandial lipids as well as oxidative stress status, thus demonstrating a remarkable anti-atherogenic effect. The elaboration of the probiotic *L. fermentum* strain ME-3 has drawn on wide international cooperative research and has taken more than 12 years altogether. The new ME-3 probiotic-containing products have been successfully marketed and sold in Baltic countries and Finland.

## Introduction

The host and its microbiota together form a complex ecological system. Despite temporary or some-times even long-lasting imbalance due to various exogenous and endogenous influences, its individual stability is still achieved by different mechanisms. However, the impaired composition of the microbiota has been associated with several health consequences, which could be avoided by regulation of the microbe and host interrelations. Probiotics aimed at stabilizing the microbial communities and the health-promoting effects have an important position in the medical health care of different age groups and diseased persons.

## Diversity and functions of intestinal microbiota

The gastrointestinal (GI) microbiota of human beings is a dynamic and complex mixture of microbes consisting of bacteria, archaea, protists (ciliates), anaerobic fungi and different bacteriophages and viruses (1). It has several diverse functions including the decomposition of different nutrients, the maturation of intestinal cells, morphology and gut physiology, stimulation of the immune system, systemic effects on blood lipids and inhibition of harmful bacteria (2). The number of microbes harboured in the human body is nearly 10 times more than the number of somatic cells: on the skin there are 10^12^, in the mouth 10^10^ and in the intestinal tract 10^14^ microbial cells. The interrelation between the host and its microbiota–both commensals and pathogens–is the subject of the microbial ecology of the human organism in health and disease.

The microbial biotope produces an environment for microorganisms with similar conditions and volume. New microorganisms are constantly arriving into the open system, yet they do not stay. During the years 1960–1970, research by R. Dubos, R.W. Schaedler and D. Savage introduced an understanding of indigenous microbial flora, which was composed from autochthonous (endogenous, specific for the host species) and allochthonous (exogenous, occasional) microbes (3–5). In addition, they recognized that two types of microbiota can be differentiated in the cavities of particular organs: the luminal and mucosal microbiota. In different microbial biotopes the mucosal microbiota is composed of the auto-chthonous microbes that have become resident due to their adhesion to eukaryotic cells via receptors or by harbouring in the mucin layer of mucosal membranes. The species composition of mucosal microbiota is individually specific and stable. In contrast, the luminal microbiota consists of some resident unattached microbes, autochthonous microbes released from different vertical biotopes of the GI tract and the transient allochthonous microbes from diet, water and the environment. The latter stay in the given biotope for a short time, then are killed or out-competed by the resident flora. The interrelations between luminal and mucosal microflora and its stability in experimental animals and humans, detected by earlier studies (6–9), have been confirmed by modern molecular methods. By using denaturing gradient gel electrophoretic analysis (DGGE), the mucosa-associated bacterial community was found to be uniformly distributed along the complete colon (10–12). However, there are also quite different understandings available: applying the fluorescent situ hybridization method (FISH) using 16S rRNA-targeted oligonucleotide probes, some authors suggest that colonic microbes are not in close contact with the mucosa and no significant differences exist between the colonic biopsies and faecal samples (13). The individual specificity (14,15) on one hand and the temporal modulation of the intestinal microbiota by different types of diet (vegetarian, western type, etc.) and stress on the other hand complicate the evaluation of different research results at the GI ecosystem level (16–18). The application of novel experimental sets (19) and sequence-based methods for the determination of microbial diversity and their functions, aptly called ‘the second Human Genome Project’, will clearly advance our knowledge of human microbial ecology (20–22).

Particular environmental microbes serve as an assumption for the individually specific composition of the indigenous microbiota of a newborn. It clearly depends on the spectrum of the mother's microbes, with which the newborn gets in close contact, on the one hand, and the individual receptor specificity/activity of its own cells, on the other hand (14,22,23). There is the possibility that the special genes of the host are involved in the determination of the particularly different characteristics of microbiocenosis. Some 20 years ago Dutch scientists showed that in the case of Crohn's disease in monozygotic twins, the quantitative composition of normal faecal microflora was genetically determined (24). Importantly, the data showed that the pattern of antibodies directed to the faecal bacteria of different morphotypes was unique for each individual, thus confirming the genetic influences of the host on their indigenous microbiota (25,26).

Using bacteriological methods, we have postulated that the quantitative composition of the faecal microflora of adult monozygotic twins has the same degree of similarity as the paired samples of a single young healthy person (8,14). The comparison of DGGE profiles in the faecal samples of monozygotic twins and unrelated persons confirmed our earlier findings on the higher similarity of the twins (22). Monozygotic twins reveal the identity of many genetic markers that are important for the selective colonization of the indigenous microflora. It is well known that both the antigenic structure of somatic cells and their secretions, as well as the immune reactivity, are determined by the genotype.

The composition of the intestinal microbiota (IMB) has been determined by several endogenous mucosal and luminal factors in the GI tract (Table I) (1,2,27–30).

**Table I:** Main endogenous factors determining the composition of the intestinal microbiota (IMB) (1,2,27–30).

1. Mucosal	2. Luminal
Specific	pH, gases
IgA	Secretion of digestive enzymes
Specificity of epithelial receptors	Peristalsis
Non-specific	Antagonism of microbiota
Complement	Lysozyme
Lysozyme	
Interferon	
Mucin	
Dendritic cells/Toll-like receptors	
Colonization resistance	

The colonization resistance (CR), defined in the early 1970s (31), is the most important first-line defence against invasive pathogenic organisms such as *Clostridium difficile, Salmonella, Shigella, Candida, albicans*, etc. and indigenous opportunistic microbes such as the urinary tract infection-causing *Escherichia coli, Proteus* sp., *Pseudomonas* sp. CR is a dynamic phenomenon influenced by the host, diet and medical interventions. Antibiotic therapy can decrease the CR some 1000-fold (2). There are several limitations in the protection by lactobacilli against the development of antibiotic-associated diarrhoea, although some association between the counts of intestinal lactobacilli and CR against *Clostridium difficile* has been found. Mainly, these depend on the species-specific antibiotic susceptibility of the individually different *Lactobacillus* spp. composition and whether or not the gut environment supports the survival of the administered probiotic lactic acid bacteria (32).

The predominant microbial species of different GI tract niches (oral cavity, stomach, jejunum, ileum and colon) are not similar, depending on the individual conditions of the biotope. However, over the GI tract the same groups of bacteria predominate, e.g. firmicutes, bacteroidetes, actino-bacteria, proteobacteria, fusobacteria and the uncultured groups (33). In the gastric cavity at physiological acidity, the number of bacteria reaches 10^3^ cfu/g, with gram-positive microbes predominating (lactobacilli and streptococci). It is well known that the number of microbes (cfu/g) increases down the intestinal tract, with anaerobic bacteria out-numbering the aerobic ones by nearly a thousand times. In the colon at different ages (10^10–11^ cfu/g), anaerobes such as *Bacteroides*, eubacteria, bifidobacteria, peptostreptococci and fusobacteria predominate over the *Lactobacillus/Enterococcus* spp., coliforms and staphylococci assessed by cultivation on selective and non-selective media (2). The cultivable anaerobic clostridia are exceptions, including *Clostridium perfringens* and *Clostridium difficile*, whose counts do not exceed 10^5–6^ cfu/g in adults (2,8). New possibilities for discovering the entity of the microbiota were achieved by the development of experimental animal studies, as well as studies on germ-free animals (34).

A 100–1000-fold difference has been found when using bacteriological and molecular methods for IMB studies; however, the crude understanding of the predominant bacteria (bacteroids, anaerobic cocci, eubacteria, bifidobacteria) according to the bacteriological studies of our laboratory remains quite similar to those obtained by molecular methods (35–40). The main difference is in some unculturable genera like *Atopobium, Clostridium coccoides* and *Clostridium leptum* groups, whose relative abundance in total count is 3.1–11.9%, 23–28% and 21–25%, respectively, estimated by the application of 16S rDNA techiques and molecular genetic probes (41–43).

As regards the other groups of bacteria, the main differences can be found in the susceptibility to environmental factors. For example, *Bifidobacterium adolescentis* is mainly detected by molecular methods due to its high susceptibility to oxygen (44) and thus can be frequently omitted in bacteriological studies.

## Impact of IMB on host metabolism

It is well known that the microbial community of the GI tract helps the host with the processing of nutrients, also producing vitamins and butyrate. The specific host–microbe interactions have been successfully studied by the estimation of different metabolites of the host, derived by microbiota. The concept of expression of the microflora-associated characteristics (MACs) in a macroorganism (45) has been further elaborated for a microbial–host crosstalk (46) having considerable value, especially when comparing the MACs with GACs, i.e. germ-free animal characteristics (34). Nowadays it has been shown that the Toll-like receptors on the different cells of the host recognize the microbiota and form the basis for the crosstalk, which can shape even the maturation and immunity of the GI cells (29,47).

Nearly 20 years ago, we found a close correlation between the counts of different groups in the IBM and some secreted metabolic compounds (volatile phenols of urine) in monocygotic twins (8,48). To date, the microbial activity can be measured using culture, labelled biomarkers, RNAs, proteins and different metabolites. The new possibilities for linking the microbial and host metabolic activities evolved with the development of new molecular/biochemical technologies (49). The genomes of several bacteria have been sequenced, elucidating the ecological success of different bacterial groups in the different parts of the GI tract. For example, *Lactobacillus plantarum* has been characterized by a very large number of phosphotransferase systems granting their success in energy accumulation, but both bacteroidetes and some bifidobacteria had an enormous number of genes involved in the utilization of complex carbohydrates (50). The human metagenome is a composite of the individual host genes and the genes present in the genomes of the trillions of microbes colonizing human bodies. Our microbial genomes, i.e. microbiome, encode metabolic capacities that we have not had to evolve on our own (51). Several new methodical approaches are in development. Transcriptional profiling (transcriptomics) can be used in experimental models to find groups of bacteria that have switched to the expression genes involved in the utilization of different exposed substances (52). Specific activity can also be analysed using a metabolomic approach to study the influence of drugs and diet on the interactions between humans and the GI tract microbiota.

Members of the 70 well-known divisions of *Bacteria* (53), such as the *Bacteroidetes* and *Firmicutes*, consist of more than 90% of all phylogenetic types in humans. Their role has remained largely unexplored; however, their balance was recently associated with the pathophysiology of obesity in animals and humans (54). It was shown that if fasting-induced adipocyte factor (*FiaF*) expression was repressed by microbes, the adipocytes increased triglyceride production. Thus, the GI microbiota also affects the energy harvest and storage (55).

Recently, we have found in elderly persons over 65 years that the intestinal *Lactobacillus* sp. is closely bound to human inflammatory and metabolic markers of blood. The count of live lactobacilli showed a close negative correlation with an important lipid metabolism marker–the oxidized LDL (oxLDL) of blood. Moreover, colonization with *L. fermentum* was negatively associated with blood glucose level, seemingly showing its high potential for carbohydrate metabolism (both hexoses and pentoses) in the gut, thus limiting its absorbance into the blood (M. Mikelsaar et al., unpublished observations). The application of particular species of probiotic lactobacilli seemingly could be a challenge for health care to control the metabolic and systemic defence reactions of the elderly, including oxidative stress-related ones. This all suggests the possibility to influence the host metabolism by modulating the intestinal microbiota by administering useful beneficial bacteria.

## Why do we need functional food and probiotics?

The disruption of the CR-granting indigenous microbiota, thus allowing potentially pathogenic microorganisms to multiply, can be attributed to a variety of illnesses. The high load of disinfectants and antibiotics is a global problem clearly associated with a more expressed imbalance of IMB among large groups of populations. This leads to an increased susceptibility to infections, and increased inflammatory, ulcerative, degenerative and neoplastic responses. Despite the wide use of antibiotics, infectious diseases remain the major cause of death from gastroenteritis in children, hospital infections due to antibiotic-resistant bacteria and recurrent infections such as urinary tract infections (*E. coli*), antibiotic-associated colitis (*C. difficile*), persistent pepticulcer disease (*Helicobacter pylori*), etc.

***For health promotion: to compete with the depletion of microbial variety, granting defence against infection and allergies; to compete with oxidative stress prone changes***.

In wealthy societies the increased stress of life, the increasing number of elderly people and reduced physical activity are considered the reasons for the large spread of civilization-associated chronic diseases such as atherosclerosis, hypertonia, tumours, diabetes, pepticulcers, neurodegenerative diseases and different syndromes such as adipositas, fatigue and depression. The crucial role of the impaired host functions is attributed to a wide consumption of processed foods that are very rich in sucrose, saturated fat and sodium. At the same time, a lot of foods are characterized by a deficiency of a number of human nutrients such as omega-3 fatty acids, arginine, glutamine, taurine, vitamins and antioxidants (56).

Artificially created environments free of bacteria contribute to the development of allergies. The important role of the IMB, particularly lactic acid bacteria, has been demonstrated in communities with different degrees of industrialization. At the beginning of the 1990s Strachan et al. (57) presented the ‘hygiene hypothesis’ to explain the differences in the industrialized and non-industrialized world. In families with more siblings and living in rural areas, there were fewer allergies than in families with just one child and living in highly hygienic conditions; this was explained in terms of the increased priming of the immune system with different infections prevalent in bigger families. However, in Australia, Patrick Holt with co-authors (58) linked the problem with an imbalance of IMB, which is the richest potential immunomodulator in an infant's organism. IMB differences were found in young children living in countries with different degrees of industrialization (59–61), mostly differing in hygienic conditions. In Estonia, infants were more often colonized with lactobacilli during the first year of life than their Swedish counterparts (Figure 1). However, 5 years after Estonia regained its independence from the Soviet system, the increased income of the people and the improvement of the food hygiene reduced the differences in the prevalence of lactobacilli between the Estonian and Swedish infants (62).

**Figure 1:**
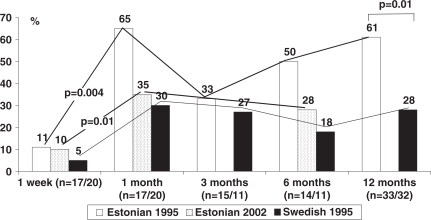
Prevalence (%) of lactobacilli among Estonian and Swedish children.

Moreover, prospective studies of an infant's colonization by indigenous microbiota in children developing or not developing allergies showed clear differences, expressed in the lowered colonization resistance of allergic children (39,40,63). The hygiene hypothesis is now revisited concerning the reduction of microbial variety in the environment resulting in fewer signals to immune system. The possibility to prevent or treat an allergy by increasing the richness of microbial communities using probiotics has attracted wide attention from researchers and practitioners (63–65).

## Probiotics – evidence-based impact on health

A probiotic is defined as a live microorganism which when administered in adequate amounts confers a health benefit on the host (66). Widely accepted probiotics contain different lactic acid-producing bacteria of human origin: bifidobacteria, lactobacilli or enterococci. The area of commensal, non-harmful bacteria of human origin serving as probiotics is rapidly expanding and in 2007 more than 3100 scientific publications were cited in the PubMed database.

Widespread agreement in understanding the probiotics area includes that the probiotic strains should be safe, effective and stable in the final product. Internationally accepted criteria have been proposed to consider the selected microbes as probiotics (66).

Yet, more importantly, up to now neither sufficient nor indicative criteria for ‘probiotic status’ have been defined. There are different *in vitro* and *in vivo* assays for testing the functional properties and the putative effectiveness of candidate probiotic strains for general health benefits or against specified diseases, recently revised by a project group Joint IDF/ISO Action Team on Probiotics (67). The search for new effective strains is an expanding process. Still, the main problem is not due to the limited discovery of new strains with new functional properties but in proving their action *in vivo*. The mechanisms granting the survival and competitiveness of the probiotics in different microbial ecosystems and action sites have not been well explored. The application of different clinical trials was the suggested clue.

The ILSI symposium (68) drafted three different levels of probiotic action: 1) direct interactions with gut microbiota, including pathogens, relying on colonization resistance mechanisms; 2) fortification of the gut barrier function by influencing the quality of tight junctions; 3) modulation of the mucosal immune cells amount and activity and the systemic immune system. Thus, probiotics normalize the composition of the intestinal microbiota and modulate the immune functions of the host. Emerging evidence has revealed that the prevention of GI tract colonization by a variety of pathogens is a primary mechanism of the beneficial effects mediated by probiotics (69–72). Besides infection control, several gut microbes have been elaborated for use as probiotics in functional food, which aims to prevent and treat various other health problems such as allergy, neoplastic growth and inflammatory bowel diseases. There are also a few sound data about the impact of probiotics on wide metabolic functions of the host. The newer areas include the influence of probiotics on the metabolism of dietary components, like lactose digestion, lipid metabolism, proteins and indigestible dietary compounds. To explore the impact of different probiotics on the cardiovascular system and lipid metabolism, important biomarkers to blood cholesterols and triglycerides, conducted only in a few well-designed clinical studies. The important area of human physiology that is relevant to functional food science according to the ILSI and FUFOSE (the European Commission Concerted Action on Functional Food Science in Europe) is, among others, the modulation of defence against high-grade oxidative stress (56).

Nowadays the concept of functional foods, including probiotic food and dietary supplements, implies their ability to beneficially influence body functions to improve the state of well-being and health and reduce the risk of disease (73). The provisional regulations of the ILSI and EU Research Commission (FUFOSE and PASSCLAIM: DOI.10.1007/s00394-005-1104-3) require sound evidence of ability to either balance and enhance the particular human functions or to reduce the risk of certain diseases through the use of probiotic microbes. Today, to define the health claims of a new probiotic, primarily two independent double-blinded, placebo-controlled studies are recommended. At the same time, the application of genomics, proteomics and metabolomics has begun to be involved in the elucidation of the mechanisms behind the interventions with useful bacteria (49). Several supporting and confounding intrinsic, ecological and technological factors may be of importance in the selection of suitable candidates for probiotics: properties of the strain; the metabolic capacity of the strain during passage through the GI tract; acid, bile and heat tolerance; and the ability to grow in milk and to metabolize different substrates, including prebiotics (32, 74–77).

The data depicted in Table II (68, 69, 78–102) show clearly that the probiotic action, including various health promotions, is strain specific, as among the probiotic preparations there are representatives of all the prevalent species of lactobacilli and their health effects are largely variable.

**Table II:** Evidence-based probiotic lactobacilli according to randomized double-blind placebo-controlled studies (adapted from 68,69).

Strain affiliation	Licensing enterprise	Published clinical evidence (reference no.)
*Lactobacillus acidophilus La5*	Chr. Hansen, Denmark	Suppression of *Helicobacter pylori* infection (78)
*L. acidophilus L1*	Campina Melkunie, Holland	Blood cholesterol-lowering effect (79)
*Lactobacillus johnsonii LA1*	Nestle, Switzerland	Suppression of *Helicobacter pylori* colonization in children (80)
*Lactobacillus gasseri OLI 27168*	Meij Milk Products, Tokyo, Japan	Suppression of *Helicobacter pylori* infection (81)
*Lactobacillus casei Shirota*	Yakult, Japan	Reduction of constipation, reduced proteolytic activity of IMB (82,83)
*L. casei* DN1 14001	Danone, France	Reduction of winter infections in elderly (84), diarrhea and respiratory infections in adults (85,86).
*Lactobacillus paracasei* LP-33	Un-President Enterprise Corp., Tainan, Taiwan	Reduction of allergic rhinitis (87)
*Lactobacillus rhamnosus* GG ATCC 53103	Valio, Finland	Reduction of diarrhoeal infections and allergy (88–92); increased expression of immune response genes (93)
*L. rhamnosus GR-1* and *L. reuteri RC-14*	Chr. Hansen, Denmark	Reduction of diarrhoeal infections in children (94)
*L. rhamnosus* HN001	Danisco, Denmark	Enhanced immunity of consumers (95)
*Lactobacillus plantarum* 299V	Probi, Sweden	Reduction of infectious complications in transplantation patients; decrease of intestinal permeability (96)
*Lactobacillus brevis CD2*	VSL Pharmaceuticals, Inc., Fort Lauderdale, FL, USA	Reduction of *Helicobacter pylori* infection, reduction of levels of polyamines in gastric mucosa (97)
*Lactobacillus reuteri* ATCC 55730	BioGaia, Sweden	Reduction of diarrhoeal infections, enhancement of consumers' immunity (98,99)
*Lactobacillus fermentum* ME-3 DSM 14241	AS Tere, Estonia	Anti-atherogenic effect due to increase of antioxidative activity indices, decrease of GSSG/GSH ratio and oxLDL of sera (100–102)

IMB, intestinal microbiota; GSSG/GSH ratio, glutathione redox ratio; oxLDL, oxidized low-density lipoprotein.

## Elaboration of probiotic *L.fermentum* ME-3 at the University of Tartu

### Short history of the scientific discovery of L. fermentum ME-3

*L. fermentum* ME-3 as an antimicrobial and anti-oxidative probiotic has been aimed at improving the oxidative stress (OxS) status of a consumer's organism and reducing the risk of enteral infections. The history of the discovery of *L. fermentum* ME-3 began in 1994, when joint research with Sweden started. The University of Linkoping (Prof. Bengt Björk-stén) initiated a scientific cooperative agreement between the Department of Pediatrics (Dr Kaja Julge, Dr Maire Vasar) and the Department of Microbiology of the University of Tartu (Prof. M. Mikelsaar) aimed at finding associations between allergies and intestinal microbiota. The University of Tartu had long-lasting experience in research into microbial ecology and the lactobacilli of the human organism (Prof. Akivo Lenzner). The faecal samples of Russian astronauts were investigated in Tartu for more than 20 years for detection of the composition of lactobacilli (103,104). Prof. B. Björksten has served as a visiting professor at the University of Tartu since 1994, teaching clinical science and advanced research principles to PhD students. For the allergy research he chose two comparative populations: Estonians with a low prevalence and Swedes with a high prevalence of allergy. Glaxo Wellcome Trust supported the study.

In this period more than 200 *Lactobacillus* strains of different species were collected (38,105). These strains have formed the basis of the culture collection of the Department of Microbiology, which is financed according to a national programme of collections in Estonia. On 2 March 1995, five *L. fermentum* isolates were obtained from a 1-year-old Estonian child (A.M., nr. 822). Paediatricians had previously confirmed the good health of the child, and it continues to be excellent at the age of 12 years. The microbiological work was performed by researchers Epp Sepp (MD, PhD), Paul Naaber (MD, PhD), Krista Lõivukene (MD, PhD), Mall Türi (Pharm. cand. med.), and senior research technician Eha-Mai Laanes. However, in this arena there could be no invention registered, as the strains of the *L. fermentum* species were also found from some other Estonian children, yet not from Swedes!

In 1996 the Dutch company MONA offered the University of Tartu an opportunity to test their collection of *L. acidophilus* strains for antioxidativite properties. The Dutch people relied on the research of Japanese authors (106) who had found, with non-standardized methods, some single antioxidative lactobacilli strains among hundreds tested. We were introduced to MONA by docent Seppo Salminen of the University of Helsinki, our research partner in the first stages of the introduction of the probiotic *Lactobacillus* GG in Finland. In turn, we invited into the research the scientists from the Department of Biochemistry (Prof. Mihkel Zilmer) who had published the first interesting papers on the markers of cellular oxidative stress (107–109). Great efforts were made to get the antioxidative markers run with bacterial cultures, as the disruption of microbial cells without any enzymatic influence was complicated and quite different from the previous work of biochemists studying the antioxidativity by methods originally developed in blood and blood cells. It was quite hard to achieve the cell-free extracts with no growth, as some intact cells of lactobacilli were always found after disruption. Then we switched to the supernatant of the disrupted lactobacilli cells, comparing this with the response of intact cells.

The cooperative work between the microbiologists and biochemists succeeded in obtaining adequate samples; however, to great disappointment, no strains with good antioxidative properties were found from the Dutch MONA collection. In contrast, among the included lactobacilli of the Estonian and Swedish children from our own culture collection, the biochemist Tiiu Kullisaar found two promising *L. fermentum* strains (strains 822-1-1 and 822-1-4, used in the laboratory under the acronyms E-3 and E-18).

Now there was a growing hope for invention! The protocols for new research projects were developed with the participation of several other microbiologists (Heidi Annuk, Reet Mändar, Jelena Štšepetova and Epp Songisepp) and biochemists (Kersti Zilmer, Tiiu Vihalemm, Ceslava Kairane and Ann Kilk).

Then, suddenly, we made a substantial mistake: an abstract on the two promising antioxidative lactobacilli strains was presented in Kiel to the congress of the International Dairy Federation. The materials of this congress were published and this paper excluded the novelty of the strains for our patent application (110). Thus, the priority was shifted for the use of the antioxidative properties in the probiotic preparations of the strains. We had to go further with only one strain to develop an innovative probiotic with tested functional properties, that was safe for consumption and effective in animal and human trials. This work has been performed in close association with clinicians of the University of Tartu and research partners from Finland and Italy. The European Research Council 5th FW offered the possibility of a joint volunteer study by the application of the synbiotic preparation (including our antioxidative strain of *L. fermentum*) with scientists of Reading University, UK (Prof. Glen Gibson); Wageningen University, The Netherlands (Prof. Willem de Vos); Lund University, Sweden (Prof. Göran Molin) and Turku University, Finland (Prof. Seppo Salminen). The results are presented below.

### Development of the probiotic L. fermentum ME-3

The development of the probiotic started according to provisional rules (73), later to some extent comprising the regulations of the FAO/WHO (66). According to WHO/FAO experts, it is necessary to properly check the strain's systematic, i.e. its identification by phenotypic and genotypic methods, and deposit it in an international culture collection. At this stage the suitable acronym ME-3 was also developed and the strain was deposited in an international culture collection (Deutsche Sammlung für Mikroorganismen und Zellkulturen, DSMZ 14241).

The origin, habitat and species of the *Lactobacillus* strains have a great impact on their value as probiotics. *L. fermentum* is a normal inhabitant of the human intestinal tract in the UK (111) and Italy (112). Some *L. fermentum* strains are able to metabolize cholesterol (113). There seems to be an age-dependent colonization by *L. fermentum*. In Greece, *L. fermentum* was not detected in children below the age of 3 years and the number of positive samples only increased with aging, reaching 72% in the elderly (114).

In 1995, the prevalence of *L. fermentum* in the Estonian group of investigated 1-year-old children (105) was also low (11%), whereas in adults and the elderly it reached 55–70% (14).

The low prevalence of *L. fermentum* as a species in young children was indirectly confirmed with an immunoblot study of 25 healthy children (A-L. Prangli et al., unpublished observations). The different protein domains of the three tested species of indigenous lactobacilli (L. *acidophilus, L. fermentum* and *L. plantarum*) induced the production of IgG antibodies with differing prevalence in the sera of control children. IgG antibodies directed to *L. fermentum* ME-3 showed a lower number of immunoreactive protein MW regions (kDa) than to *L. plantarum*, whose species has been more frequently detected (33% vs 11%) than the former among the microflora in children (105). This finding correlates well with the results of previous studies showing that different *Lactobacillus* spp. could possess different immune system activating properties (26,115,116) that may also be related to their colonization ability in different people.

It is well known that the main microbes of the normal microbiota of a child are obtained during labour from the mother's vagina and perineum, and from skin, breast milk and the maternity hospital environment. *L. fermentum* is a frequent inhabitant of the vagina (14). The successive colonization with a mainly gram-positive microbiota is speeded up if the child is breast-fed and in close contact with its mother (36). Afterwards, with the introduction of solid food the microbiota becomes more diverse. However, up to the second year of life an infant's microbiota is still different from an adult's as regards some groups of bacteria and their metabolic activities; yet it starts to more closely resemble the adult's microbiota (117). Seemingly, the maturation of the host immune and receptor systems and differences in consumed food are responsible for the usual discovery of *L. fermentum* in adults and not in young children. Thus, a child originally harbouring the ME-3 strain could be considered an exceptional one.

### Proper identification

The probiotic strain ME-3 was identified according to its morphological (Figure 2) (41, 118, 119) and cultural properties, negative catalase test, produces lysozyme, gas from glucose and NH_3_ from arginine, and the acidity in milk is relatively low (1.07%). According to the API 50 CHL kit (BioMerieux, France) analysed by API Lab Plus softwear, very good identification rates were obtained (ID 99.6%, T 0.87, only one test against). The strain is able to ferment ribose, galactose, D-glucose, D-fructose, D-mannose, aesculin, maltose, lactose, melibiose, saccharose, D-raffinose, D-tagatose and gluconate (118, 120).

**Figure 2:**
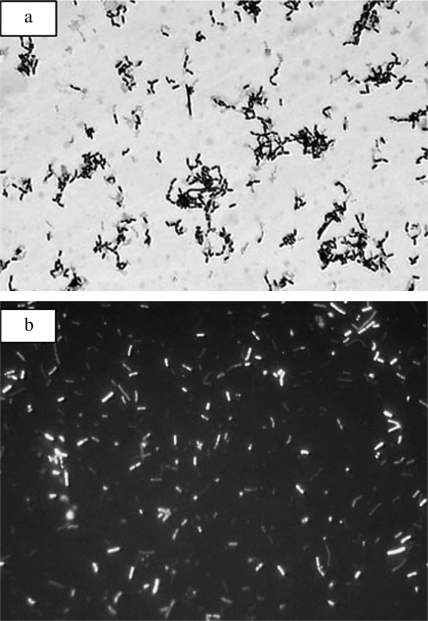
*Lactobacillus fermentum* ME-3 (DSM 14241) (118, 119). (a) Light microscopy, Gram stain, magnification ×1000. (b) Fluorescent *in situ* hybridization (FISH). Probe: Lab 158, Lactobacilli+enterococci according to Franks et al. (41).

The internal transcribed spacer polymerase chain reaction (ITS-PCR) followed by enzymatic restriction was used to confirm the species identification of the strain as *Lactobacillus fermentum* (105, 118). According to the changes in the previous taxonomy, the II biotype of *L. fermentum* has now been given distinct species status – *L. reuteri* with type strain DSM 20016, isolated from humans showing the close relationship of these two species (69,99). Further, in the patenting process in the US, it was necessary to prove the difference between ME-3 and the *L. reuteri* strain RC-5 (previously assessed as *L. fermentum* RC-5 (69)). We used ITS-PCR followed by restriction by the *Taq* I enzyme and compared the restriction banding pattern with the reference strains of *L. fermentum* ATCC 14931 and *L. reuteri* DSM20016) (Štšepetova, laboratory protocol).

Further, the fingerprints of strain ME-3 (by arbitrarily primed PCR (AP-PCR) with two primers ERIC1 and ERIC2, DNA Technology A/S, Aarhus, Denmark) were compared with some other *L. fermentum* strains (Figure 3), important in recognizing the strain in different materials such as faeces and functional food (119–121).

**Figure 3:**
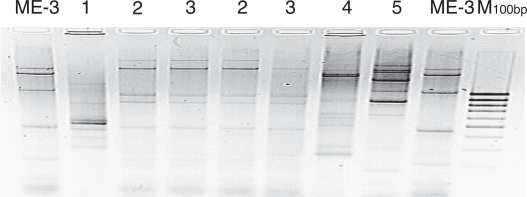
AP-PCR fingerprints for different *L. fermentum* strains. The two lanes of strain ME-3 have been generated using two DNA samples that were extracted with a time interval of 6 months. Lanes 2 and 3 contain DNA of *L. fermentum* strains isolated from the same person. Lane M contains a 100 bp DNA ladder.

In the Dutch laboratory of Wageningen University (Professor Willem de Vos), the EU Marie Curie stipend Jelena Štšepetova succeeded in showing the position of strain ME-3 in the phylogenetic tree of *Lactobacillus* spp. (Figure 4) by sequencing the 16S rRNA of strain ME-3, cloning it into the *Escherichia coli* strain, sequencing using the Sanger method and constructing the phylogenetic tree (J. Štšepetova, unpublished observations).

**Figure 4:**
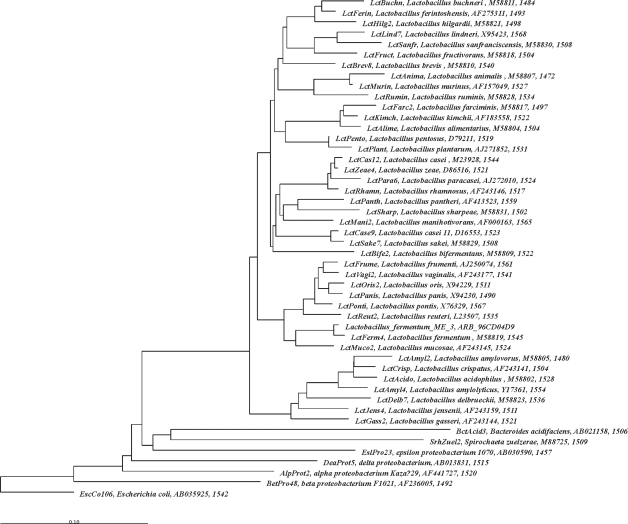
Phylogenetic tree based on 16S rRNA sequencing showing the relationship of *L. fermentum* ME-3 to the closest related lactobacilli. Analysis was performed with the ARB software package.

### Metabolic activity

According to the systematics developed by Kandler and Weiss (122), the strain ME-3 belongs to the obligately heterofermentative lactobacilli with a characteristic type of metabolism. We have determined the metabolites of *Lactobacillus* sp. by the gas chromatographic method using the Hewlett-Packard model 6890 of gas chromatography (Figure 5) (123).

**Figure 5:**
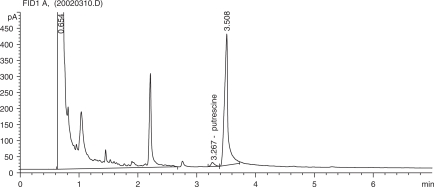
Gas chromatography of polyamines of *L. fermentum* ME-3 in the decarboxylation medium with ornithine (123).

The metabolism (Table III) was dependent on the environment, whereas in an anaerobic environment as compared to a microaerobic one, much more ethanol and succinic acids were produced (121).

**Table III:** Production of short chain fatty acids (SCFAs), ethanol, polyamines, conjugated linoleic acid (CLA) and nitric oxide (NO) by *L. fermentum* ME-3 DSM 14241 in comparison with *Lactobacillus plantarum* DSM 21380.

Indices*	ME-3	DSM 21380^1^
Short chain fatty acids (mg/ml) 24–48 h incubation		
Lactic acid	10.6–11.1	10.1–11.6
Acetic acid	0.8–0.9	0.08–0.1
Succinic acid	1.8–1.9	0.07–0.07
Ethanol	9.8–7.5	0
Polyamines (μg/ml) 96 h incubation		
Decarboxylation of arginine		
Putrescine	0	0
Cadaverine	0.6	0
Decarboxylation of glutamine		
Putrescine	0.8	0
Cadaverine	0.5	0
Decarboxylation of ornithine		
Putrescine	1.3	0.5
Cadaverine	0	0.6
Conjugated linoleic acid (mg/l)	12.4	39.9
NO production (μM)	1.2±0.2	2.6±0.8
H_2_O_2_ production (μM)	484±200	196±129

* Microaerobic environment.

Recently we have discovered that strain ME-3 is able to produce nitric oxide (NO) in MRS medium (Table III). NO production was assessed by the Apollo free radical analyzer 400 in MRS fluid media (Oxoid, UK) after incubation for 48 h. The NO is able to induce among others the protection against inflammation, while in an ischaemic heart the NO can functionally activate the cellular anti-oxidant defence systems (124).

In the decarboxylation medium (125) containing amino acids such as arginine, ornithine, lysine and histidine, it was possible to assess the production of the polyamine putrescine and minimal amounts of biogenic amine cadaverine (<1 μl/ml) by ME-3. This amount is really minimal compared with *E. coli*, which produced significantly larger amounts of cadaverine (240 μl/ml) in the lysine media.

The metabolism of the putrescine is closely connected with organic acids metabolism, as in gut putrescine may be converted into the acetylated and oxidated succinate. Besides, it is known that some polyamines can serve as antioxidants (126).

When strain ME-3 was grown in milk media for 4, 10, 20, 30 and 40 days, no biogenic amines were detected, confirming the safety of strain ME-3 for the production of functional food (123).

### Acid and bile tolerance

The definition of probiotic implies that the bacteria should be viable at the time of ingestion and after passage through the GI tract. This should be achieved by the ability of a particular probiotic strain to tolerate the highly acidic conditions present in the stomach and the concentrations of intestinal juices and bile salts found in the small intestine. Several *in vitro* assays, including tolerance to the harsh conditions of the digestive tract and adhesion to the host's gut epithelial cells, have been used to demonstrate the survival of the probiotic strain inside the gut (127). Usually after oral administration the expected results include faecal recovery of the strain and correction of the imbalance of intestinal microflora by the probiotic (128, 129). However, it has not been assessed if the improvement of the microbial balance in the upper part of the digestive tract should always be expressed in the abundant microbial communities of the large intestine and faecal samples. In experimental animal studies, it has been shown that there was quite a few association (<30%) between the mucosal flora of the upper parts of the gastrointestinal tract and the fecal microflora (23, 35). Pereira and Gibson (113) have thoroughly studied the degree of acid and bile tolerance of the human isolate *L. fermentum* KC and have shown its ability to maintain viability for 2 h at pH 2 and to grow in a medium with 4 mg of bile acids per litre.

*L. fermentum* ME-3 was found to tolerate the drop of pH values from 4.0 to 2.5 without a loss in viable cell count. Even at pH 2.0 the strain survived for 6 h and only after that the numbers of viable cells fell rapidly (118). Strain ME-3 tolerated all the tested bile concentrations (0.3–2.0%) similarly well during 24 h without any remarkable loss in viable counts. By the co-action of pH and pepsin followed by bile and pancreatin, a decrease of 0.5 log to 1.5 log of viable count was noticed. Also, the producer of strain ME-3 freeze-dried culture (Probiotical OY, Novara, Italy) has confirmed the tolerance of strain ME-3 to harsh environmental conditions. Besides, the high stress tolerance of strain ME-3 in harsh GI tract conditions after a 3-week consumption of goat milk fermented by the addition of strain ME-3 was shown (Figure 6) by confirmation of strain ME-3 in faecal samples of 100% of volunteers by molecular methods (101).

**Figure 6:**
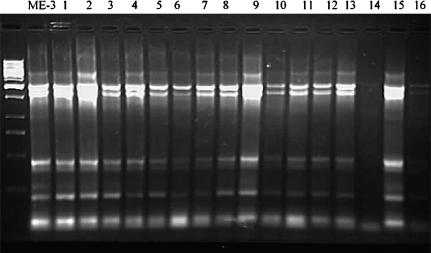
Recovery of *L. fermentum* ME-3 in faecal samples of all volunteers after consumption of ME-3 fermented goat's milk.

### Lectin typing

The carbohydrate pattern of ME-3 cell surfaces was tested with different lectins in the laboratory of Professor Torkel Wadström (University of Lund, Sweden). Lectins are oligomeric and multimeric plant or animal proteins or glycoproteins with binding specificities toward a particular carbohydrate structure. Multimeric structure gives lectins the ability to agglutinate cells or form precipitates with glycoconjugates (130). The monosaccharides such as N-acetylglucosamine, N-acetylgalactosamine and mannose are present in the glycocalyx of lactobacilli in significant amounts in the typical gram-positive envelope (131). These sugars have been linked to adhesive properties of lactobacilli. *L. fermentum* ME-3 whole cells, similar to other tested lactobacilli strains of the same species, showed specificity to D-Gal, D-GalNAc carbohydrates by lectin *Bandeirarea simplifolica* I (132). Harbouring this profile seemingly helps strain ME-3 to compete with *E. coli* for adhesion to gal-gal receptors in the intestinal tract, where *E. coli* some virulent strains can be reservoir for recurrent urinary tract infection.

We have seen some effect in a clinical trial for preventing this recurrent infection in children. The data in the literature showed that re-infection with a new uropathogenic *E. coli* residing in the intestinal tract occurred in nearly 75% of adult women (133). We wondered if colonization with strain ME-3 could suppress the potentially pathogenic *E. coli* and decrease the recurrent episodes. *In vitro* experiments were promising, as the *E. coli* ATCC strains and several clinical isolates were suppressed on culture media for 2–3 log cfu/g. We hoped that this could decrease the load of uropathogenic *E. coli* in the intestinal tract to prevent new recurrent episodes of urinary tract infections. Children who had experienced a first attack of acute pyelonephritis consumed either strain ME-3 or a placebo for 3 months: in the placebo group, the recurrence rate was 50% while in the strain ME-3 group it was a somewhat lower 32% (Vainumäe, dissertation in preparation).

### Antimicrobial activity

The prevention of GI tract colonization by a variety of pathogens is a primary mechanism of beneficial effects mediated by probiotics (70,71). It has been shown that the large spectrum of different metabolites is responsible for the suppression of the growth of pathogens *in vitro* and for their competitive exclusion in animal models. Many of the metabolites produced by lactic acid bacteria have a broad antimicrobial activity against some other species, especially gram-negative ones, in contrast to particular bacteriocins that usually inhibit only closely related species among other gram-positive bacteria (134).

*L. fermentum* ME-3 has the ability to suppress mainly gram-negative bacteria but to some extent also enterococci and *Staphylococcus aureus*. Its antagonistic properties against enteral pathogens (*Salmonella* Typhimurium and *Shigella sonnei*), and urinary tract infections caused by *E. coli*, were assessed by a streak line procedure on plates containing modified (pH 7.2) MRS medium (135–137). In different environmental conditions (microaerobic and anaerobic milieus) the production of lactic acid by strain ME-3 correlated well with its antagonistic activity.

Beside lactic acid, the acetic and succinic acids and ethanol produced in substantial amounts by strain ME-3 in microaerobic conditions, the production of NO and H_2_O_2_ could be responsible for the antimicrobial effect (Table III). In her PhD dissertation, Heidi Annuk was able to construct a diagram showing that the *in vitro* antagonistic activity, seemingly due to a pH drop and organic acids production, was quite characteristic of particular fermentative groups of lactobacilli (homo-, facultatively heterofermentative and obligately heterofermentative) further identified by ITS-PCR (137).

Recently, we have found by the ROS analyser (APOLLO 4000) that the ratio of signals H_2_O_2_:NO was 13.7, produced by strain ME-3 in MRS medium, achieving the first rank among about 30 tested strains of *Lactobacillus* species. This shows that strain ME-3 is able to manage with both compounds to suppress antagonists and/or initiate signalling using several pathways (Table III).

Remarkably, the modest antimicrobial activity of *L. fermentum* ME-3 (138) expressing some antimicrobial cationic peptides against the *Helico-bacter pylori* reference strain NCTC 11637 was changed for high activity against clinical *H. pylori* isolates (139). So far, the most effective management of *H. pylori* infection causing gastritis and pepticulcer disease is combined antimicrobial therapy. Some authors have applied the antagonistic effect of lactic acid bacteria against *H. pylori* in the prevention of and therapy for *H. pylori* infection (140), although some failures are also described.

Seemingly, a different tropism of the pathogen and probiotic, besides the inadequacy of the dosage and intervention, survival of the probiotic in the gut and *Lactobacillus* spp. differences in antagonistic activity and immunomodulating properties, could emerge among putative reasons for the low efficacy of probiotic therapy in GI infections (141). Moreover, the main mechanisms of probiotic action–such as competing for nutrients, producing antimicrobial substances, blocking the adhesion and toxin receptor sites, removing it by co-aggregation, stimulating the immunity, increasing the barrier function and the attenuation of virulence–have to be differentially expressed in extra- and intracellular infections, and in fact, they are rarely all present in a particular probiotic.

Thus, strain ME-3 possesses several antimicrobial characteristics such as acetic, lactic and succinic acids, putrescine, NO, CO_2_ and H_2_O_2_, produces some cationic peptides, has a suitable lectin profile for competitive adhesion to the epithelium and some immunogenic properties, as assessed in animal experiments and human studies.

### Safety

First, lactobacilli and bifidobacteria are historically considered safe for their close association with food. Second, they are inhabitants of the normal indigenous microbiota and their pathogenic potential is low. However, some *Lactobacillus* spp. strains have been associated with systemic and local infections (142). They can cause a problem in a world with an increased immunocompromised population. Laboratory investigations, experimental animal studies and volunteer trials were conducted to test the safety of the ME-3 strain. The *in vitro* experiments confirmed the absence of haemolysins and the suppression of indigenous lactobacilli and bifidobacteria, tested in co-cultivation experiments with particular strains.

The most important feature for safety assurance suggested by a large EU study was the absence of transmissible antibiotic resistance genes/plasmids and the natural resistance to trimethoprim plus sulfamethoxazole, metronidazole, fluoroquinolones and cefoxitin that corresponded to the wild strains of the *L. fermentum* species (143).

The safety of ME-3 has been tested repeatedly in a mouse model (NIH line conventional male mice, Kuopio Finland) administered commercial diet R-70 (Lactamin, Sweden). The experiments were performed according to European Convention regulations for animal experiments no. 123 from 1986. Freeze-dried *L. fermentum* ME-3 was added to the tap water in a daily dose of 9.7 log cfu for 30 days. The mice were monitored and the faeces were collected individually for the detection of strain ME-3 daily. All strain ME-3-challenged animals remained in a good state during the feeding trial. Furthermore, safety was also confirmed by feeding mice during several months with a probiotic ME-3 cheese in different doses (136).

The mice were killed by cervical dislocation and autopsies were performed under sterile conditions using a Class II microbiological safety cabinet (Jouan, France). To estimate the content of lactic acid bacteria in the terminal ileum and colon, bacteriological investigations were carried out immediately. No translocation of the probiotic strain into any organs (ileum, liver and spleen) was detected. The increase (from 8.6±0.3 before to 9.1±0.2 log cfu/g after the trial, *p* = 0.003) in total faecal lactic acid bacterial counts was observed only at the end of the 30-day experiment (Truusalu, dissertation in preparation).

## Antioxidative effects

### Survey of oxidative stress and antioxidative effects

Oxidation is essential to living organisms for energy production. However, it is now well established that abnormal formation of the reactive species (including free radicals) occurs *in vivo* and can lead to the damage of lipids, proteins, nucleic acids and carbohydrates of cells and tissues (144). In the human body reactive oxygen species (ROS) and reactive nitrogen species (RNS) are the main players. Both terms involve appropriate free radicals and non-radical species A free radical is defined as an atom or molecule having at least one unpaired electron in its outer orbital; examples include oxygen-centred radicals (superoxide radical, hydroxyl radical), nitrogen-centred radicals (nitric oxide, nitric dioxide radical), sulphur-centred (RS^−^ thiyl), and some others. Principal ROS are superoxide radical, hydroxyl radical, lipid peroxyl radical and non-radical hydrogen peroxide (the latter is produced from superoxide by superoxide dismutase). Principal RNS are nitric oxide and non-radical peroxynitrite. The pathological efficiency of the hydroxyl radical is the most potent and it is rapidly generated via the Fenton cycle where free iron (a very potent prooxidant) reacts with hydrogen peroxide (144).

ROS and RNS are generated within the body from different external reactions (radiation, pollutants, toxins, chemicals and drugs) and internal univalent biochemical redox reactions. Several diseases are associated with the toxic effect of the transition metals (iron, copper and cadmium). An excessive production of reactive species leads to an imbalance in the prooxidants–antioxidants system. Any imbalance in favour of the prooxidants potentially leading to damage was termed ‘oxidative stress’ (145).

There is a large body of evidence that high grade oxidative stress (OxS) has one of the crucial roles in the pathogenesis of several disorders/diseases of the GI tract (inflammatory bowel disease, coeliac disease, etc.), the vascular system (atherosclerosis, myocardial infarction, stroke, vascular dysfunctionality), the nervous system (Alzheimer's disease, Parkinson's disease), the liver (ethanol damage, cirrhosis), the skin (several dermatoses), the pancreas (diabetes mellitus) and the eyes (age-related macular degeneration, retinopathy) (144–152). Metabolic syndrome, obesity, premature ageing and the development of several tumours also have an OxS-related background (153).

Atherosclerosis is characterized by many potential risk markers such as increased values of low-density lipoprotein (LDL)-cholesterol and elevated blood pressure, while HDL-cholesterol, fasting triacylglycerol and plasma homocysteine are suggested as the diet-related markers (154). Moreover, the increased inflammatory markers (white blood cells (WBCs), highly sensitive C-reactive protein (hsCRP)) and the oxidative stress indices (oxidized LDL, urine 8-isoprostanes, etc.) have been shown to be characteristic of patients with atherosclerotic lesions of the vascular system, having developed cardiovascular disease (CVD) (146).

For the systemic multi-level control of OxS, the human body has evolved an integrated antioxidant defence system (Figure 7). It includes both the non-enzymatic antioxidants like reduced glutathione (GSH) and vitamins E, C and Q_10_, blood albumin, uric acid, bilirubin and enzymatic antioxidants like superoxide dismutase (Cu, Zn-SOD, Mn-SOD), glutathione peroxidase (GSHPx), catalase (CAT) and haem oxygenase (144).

**Figure 7:**
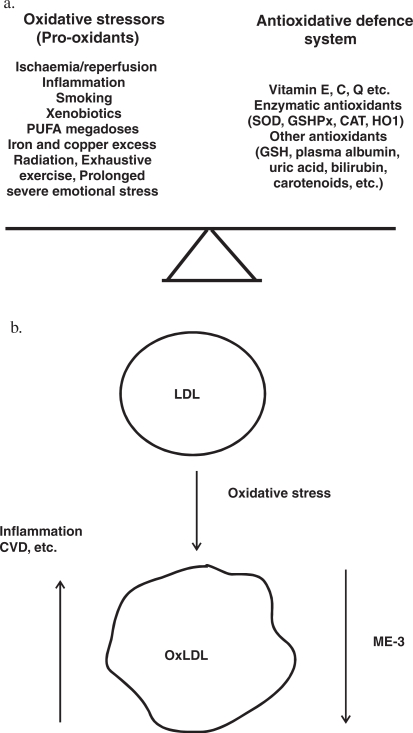
A net of prooxidants and the potency of antioxidant defence system normally balanced in the human body. (a) A summary effect of oxidative stressors and potency of antioxidant defence system of the human body are normally balanced. An imbalance leads to oxidative stress. PUFA, polyunsaturated fatty acids; SOD, superoxide dismutase; GSHPx, glutathione peroxidase; CAT, catalase; HO1, haem oxygenase; GSH, reduced glutathione. (b) Oxidative stress causes the production of oxidized LDL (oxLDL), which is a potent atherogenic and inflammatory agent. Strain ME-3 lowers the level of oxLDL. LDL, low-density lipoprotein; CVD, cardiovascular diseases.

Several antioxidative components that are integrated into the human antioxidant defence system are derived from foodstuffs and/or provided by GI microbiota. However, up to the mid-1990s a few studies (106) had been carried out to screen for the antioxidative properties of indigenous microbiota, including bifidobacteria and lactobacilli. Furthermore, it became apparent that the integrated antioxidant defence system and GI microbiota of the human body are very tightly linked, whereas specific strains with physiologically effective antioxidative properties may have a great impact on the management of the OxS level in the gut lumen, inside mucosa cells and in the blood, to support the functionality of the integrated antioxidant defence system of the human body.

Some new trends for medical bioremediation target the application of microbial catabolic diversity against aging and several major age-related diseases such as atherosclerosis. This follows the historical hypothesis of Metschnikof suggesting the intensive consumption of lactic acid bacteria in order to postpone aging (cited in Vaughan et al. (155)). However, it was not assessed whether the increased content of lactobacilli and temporal colonization with their particular strains could influence the inflammatory and OxS-derived blood indices. We have tried to explore this gap in our probiotic studies.

## Studies on the antioxidative effect of *L. fermentum* ME-3

### In vitro studies

In 1996 we started to check the antioxidative characteristics of a large number of *Lactobacillus* spp. strains. Applying a number of different tests we found that two strains of *L. fermentum* may have an impact as new probiotics with functional properties towards antimicrobial and antioxidative action. Both strains (E-3 and E-18) and their lysates had physiologically relevant multivalent antioxidativity (TAS and TAA test) to overcome the exogenous and endogeneous OxS of the host (156). The antioxidative properties of *L. fermentum* ME-3 and demonstrated effects are summarized in Table IV (100–102, 136, 156–164, 167).

**Table IV:** Antioxidativity-related properties and effects of strain ME-3.

Property/effect	Experimental (ES), animal (AS), human (HS) study (reference nos)
Expression of Mn-SOD, prolonged survival time in presence of high H_2_O_2_, scavenging of superoxide and hydroxyl radicals	ES (156)
Characterized by high TAA and TAS values	ES (156,157)
Containing of GSH and related antioxidative enzymes	ES (157,158)
Working as natural antioxidant in soft cheese spreads with different fats	ES (159)
Maintaining its high TAA during production of probiotic cheese	ES (136)
Removal effect of metals (prooxidants) from environment	ES (160)
Elevation of blood TAS or TAA and TAA in the gut mucosa	HS, AS (100,102,157,161,162)
Elevation of oxyresistance of LDL	HS (100,157,162)
Lowering level of oxLDL	HS (100,102,162)
Lowering level of isoprostanes	HS (100,162,163)
Lowering the glutathione redox ratio in blood, in the gut mucosa, in skin	HS, AS (100,102,157,161,164)
Lowering lipid peroxidation in the gut mucosa	AS (161,164)
Lowering level of BCD-LDL	HS (100,162,167)
Positive effects on post-prandial status of OxS, blood lipoprotein status and urine isoprostanes	HS (162,163)

BCD-LDL, baseline diene conjugates in low-density lipoprotein; GSH, reduced glutathione; H_2_O_2_, hydrogen peroxide; LDL, low-density lipoprotein; Mn-SOD, manganese superoxide dismutase; oxLDL, oxidized low-density lipoprotein; OxS, oxidative stress; TAA, total antioxidative activity; TAS, total antioxidative status.

Researcher Ann Kilk of the Department of Biochemistry performed PCR for the detection of manganese superoxide dismutase (Mn-SOD) in ME-3 cells. Mn-SOD is very important in the control of lipid peroxidation. Manganese and Mn-SOD activity of lactobacilli (not having a usual catalase) is important for their survival in the oxidative milieu (milk, host) created by the production of H_2_O_2_ (165, 166).

Furthermore, researcher Tiiu Kullisaar designed an original set of experiments showing that strain ME-3 (its previous in-laboratory acronym was E-3) had a good hydroxyl radical scavenging efficiency and was able to survive in high concentrations of hydrogen peroxide content and superoxide anions quite similarly to a highly ROS-resistant *Salmonella* Typhimurium, although lactobacilli did not have a catalase as compared with salmonellas (156). The survival in the presence of different ROS was possible due to GSH that is a major non-enzymatic intracellular antioxidant and protector-molecule (144). Recently it was established that ME-3 cells possess a complete glutathione system for its synthesis, uptake and redox turnover (158).

An independent laboratory confirmed that the *in vitro* superoxide anion scavenging efficiency of strain ME-3 was more than 80–100 times stronger as compared with trolox or ascorbic acid (Ahotupa, personal communication). That the antioxidant properties of strain ME-3 prevented the oxidative spoilage of soft cheese products has also recently been confirmed by experiments in Finland (159).

### Experimental animal infections

Next, an animal model of typhoid fever was developed by the inoculation of mice with *Salmonella* Typhimurium (161). *S*. Typhimurium induces generalized infection in mice with typhoid nodules (granulomas) in the liver and spleen.

In the prophylactic and treatment model with an *S*. Typhimurium challenging of mice, the application of *L. fermentum* ME-3 was not able to eradicate salmonellas from organs, yet strain ME-3 suppressed the excessive OxS-related reactions caused by the infectious agent and the inflammation. The enhanced lipid peroxidation and the abnormal glutathione redox ratio were corrected and thus the gut mucosal antioxidative status was improved (161).

The still wide prevalence of typhoid fever in southern countries and the treatment problems have got our attention. Therefore, we have applied the same experimental typhoid fever model for the elaboration of the treatment principles of *S*. Typhimurium infection with an antimicrobial quinolone (ofloxacin) combined with the probiotic ME-3. The combinations of antimicrobial preparations with probiotics have been used with good success for the treatment of *H. pylori* infections, yet mainly to cope with adverse effects (140). We succeeded in showing the priority of the combination for the eradication of salmonellas from the intestinal tract and liver (Figure 8) and also the reduction of typhoid nodules (granulomas) in the liver (Figure 9).

**Figure 8:**
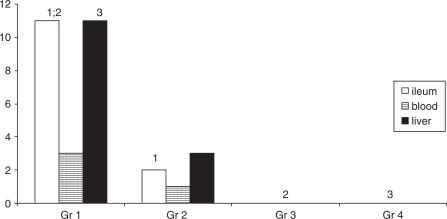
The number of mice with viable *Salmonella* Typhimurium in ileum, blood and liver. Gr1, *Salmonella* Typhimurium (ST)-challenged mice; Gr2, ST treated with ofloxacin (OFX); Gr3, ST treated with strain ME-3; Gr4, ST treated with OFX + strain ME-3. ^1^*p* = 0.032 Gr1 vs Gr2 ST in ileum; ^2^*p* = 0.002 Gr1 vs Gr3 and Gr4 ST in ileum; ^3^*p* = 0.002 Gr1 vs Gr3 and Gr4 ST in liver.

**Figure 9:**
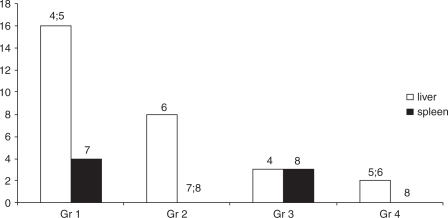
The number of mice with typhoid nodules in liver and spleen. ^4^*p* = 0.027 Gr1 vs Gr3; ^5^*p* < 0.001 Gr1 vs Gr4; ^6^*p* = 0.023 Gr2 vs Gr4; ^7^*p* = 0.002 Gr1 vs Gr2 and Gr4; ^8^*p* = 0.048 Gr2 vs Gr3 and Gr3 vs Gr4.

Meanwhile, the indices of lipid peroxidation were reduced (Table V) and the glutathione redox ratio (GSSG/GSH) was significantly lower in mice treated with the combination of strain ME-3 and ofloxacin (164).

**Table V:** Indices of oxidative stress (with standard deviations) in the ileum mucosa in mice challenged with S. Typhimurium and treated with ofloxacin and/or the probiotic *L. fermentum* ME-3.

Experimental groups	LPO (pmol/mg protein)	GSSG/GSH
*Salmonella* Typhimurium challenged mice (Gr1)	338±46^1^;^4^	0.26±0.41^3^;^5^
ST treated with ofloxacin (OFX) (Gr2)	228±41^2^	0.26±0.11
ST treated with strain ME-3 (Gr3)	169±11^1^;^2^	0.16±0.20^3^
ST treated with OFX + strain ME-3 (Gr4)	161±27^1^;^2^	0.17±0.11^3^
Control (PBS)	157±24^4^	0.11±0.2^5^

GSSG/GSH, glutathione redox ratio; LPO, lipid peroxides; OFX, ofloxacin; PBS, phosphate-buffered saline; ST, *Salmonella* Typhimurium.

1 *p* < 0.001 Gr1 vs Gr3 and Gr4;

2 *p* = 0.002 Gr2 vs Gr3 and Gr 4;

3 *p* = 0.006 Gr1 vs Gr3 and Gr4;

4 *p* < 0.001 Gr1 vs control;

5 *p* < 0.003 Gr1 vs control.

Our last *in vivo* experiments on the NIH mouse model showed that the administration of strain ME-3 induces high levels of anti-inflammatory cytokine interleukin (IL)-10 in both the gut and liver tissue. This could be the reason for the reduced number of typhoid nodules in the liver in mice treated with a combination of ofloxacin and strain ME-3 (164).

Thus, strain ME-3 helps to alleviate OxS- and inflammation-related disorders in the intestinal cells in different ways (cf. discussion about ME-3 action mechanisms).

### Volunteers and clinical trials

Strain ME-3 colonization and safety, as well as its antioxidative effects, have been tested in several open placebo-controlled and randomized double-blind placebo-controlled clinical trials (100–102,162,167) using capsules with ME-3, goat milk fermented with ME-3, commercial foodstuffs (kefir, cheese) and synbiotics enriched with ME-3. A large spectrum of indices measured in healthy adult volunteers showed that the use of strain ME-3 was safe regarding the physiological values of blood cytokines (including IL-6), inflammatory markers (WBCs, hsCRP), principal markers of carbohydrates and lipids or lipid-like compounds (glycose, triglycerides, cholesterol, LDL, HDL), several metabolites (homocysteine, creatinine, bilirubin) and several other biochemical indices such as blood calcium and iron, and en-dothelial functionality and arterial stiffness.

The consumption of strain ME-3 had a positive influence on the gut microbiota. The faecal counts of beneficial lactobacilli were increased in several studies (Figure 10) offering protection against colonization by potential pathogens if the volunteers consumed goat milk fermented by strain ME-3 (daily dose 3 × 10^11^ cfu) or used freeze-dried capsu-lated ME-3 (10^9^ cfu per capsule twice a day) as well for 3 weeks. In contrast, in the group of volunteers consuming non-fermented goat milk, a decrease in total lactobacilli counts was seen during the 3-week trial. However, a significant difference was found concerning the applied formulations. In the goat milk trial strain ME-3 was molecularly (ITS-PCR) assessed in all volunteer consumers, yet strain ME-3 was not detectable among ME-3 isolates by bacteriological methods or by AP-PCR in the probiotic capsule trial, although there was a positive shift in blood indices. In capsulate forms higher quantities of bacteria (up to 10^10^ cfu/g) have been suggested to re-isolate the probiotic bacteria from faecal samples (168,169).

**Figure 10:**
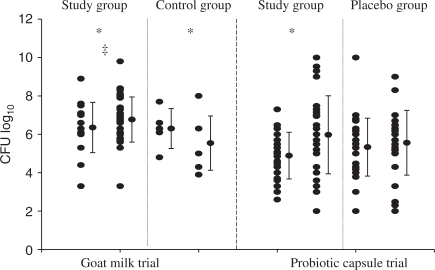
Increase of total faecal counts of lactobacilli in healthy volunteers consuming strain ME-3 in fermented goat milk and in the DBRP probiotic capsule efficacy trial (118). *p <0.005 difference from pretreatment values; ‡p <0.01 difference between ME-3 and control treatments.

At the same time, the consumption of ME-3 bacteria-containing substances showed positive effects on several OxS-related indices such as postprandial lipid, lipoprotein and OxS profile of blood and urine (162). Consumption of kefir containing strain ME-3 significantly decreased the postprandial content of fats, oxLDL and BDC-LDL, enhanced the level of HDL and improved the bioquality of HDL particles.

#### Synbiotic trial

*L. fermentum* ME-3 was also applied in the EU Research Commission-funded project ‘EU and Microfunction–Functional assessment of interactions between human microbiota and host’ (QLRT-2001-00135; ISRCTN43435738) using a randomized, double-blind crossover synbiotic intervention study with 53 healthy volunteers (167,170). The composition of the synbiotic was as follows: probiotics *L. fermentum* ME-3, *L. paracasei* 8700:2, *Bifidobacterium longum* 46 (both Probi, Sweden) and 5 g of prebiotic Raftilose P95 (Orafti, Belgium). Several shifts in the microbiota composition were assessed by research partners at Reading University (UK), such as an increase in bifidobacteria and clostridia counts, associated with appropriate shifts in profiles of faecal metabolites (short chain fatty acids, SCFAs). An increase in the total antioxidative activity was also registered (Table VI) accompanied by an improved bioquality of LDL particles (oxLDL and BDC-LDL) of sera of volunteers after consumption of the symbiotic for 3 weeks.

**Table VI:** Improvement of OxS-related indices of blood sera in the synbiotic DBRP crossover study in healthy volunteers (167).

Blood indices	Baseline	Final synbiotic	Paired *t* test
TAA%	41±2	42±2	<0.001
oxLDL (ApoB-modified)	132.5±50.5	122.8±45.6	0.047
BDC-LDL (diene conjugates)	15.2±6.1	12.7±4.1	<0.001

BCD-LDL, baseline diene conjugates in low-density lipoprotein; oxLDL, oxidized low-density lipoprotein; TAA, total antioxidant activity.

However, half of the healthy asymptomatic volunteers in this trial were colonized with *H. pylori*. All applied probiotic strains expressed a high antagonistic activity against the *H. pylori* reference strain *in vitro* (138). Despite the consumption of the symbiotic for 3 weeks, this composition could not eradicate the *H. pylori* infection (167). Seemingly the application of the entero-coated probiotic capsules prevented the direct effect of lactobacilli on *H. pylori* on gastric mucosa. Thus, the antagonistic bacteria, including strain ME-3, were not able to exert any systemic antimicrobial influence on *H. pylori* colonization, although the same consumed composition improved the antioxidative indices of blood in persons colonized with *H. pylori*.

#### Special clinical trials on patients

On the basis of the information about safety and the positive effects of strain ME-3 in healthy volunteers, we also conducted some preliminary clinical pilot trials.

In the first randomized blinded pilot study, patients with mild to moderate atopic dermatitis consumed goat milk fermented with strain ME-3 for 3 months (102). The clinical SCORAD index decreased from 4.8±3.9 to 1.9±1.8 (*p*<0.05) in the probiotic group and from 4.8±2.8 to 2.3±0.9 in the control group. A significant amount of oxidized iron (prooxidant) was found in the skin of patients before consumption and the values of iron were decreased. Also, the diene conjugate values (indicating lipid peroxidation) were reduced (Figure 11). In the same group, the antioxidativity markers of blood also showed an improvement. The levels of oxLDL decreased, and GSH (a protective form of glutathione) levels increased with a concomitant reduction in the GSSG/GSH ratio both in the skin and in the blood level. The results of our study demonstrated that the regular use of probiotics with antioxidative properties decreased inflammation and concomitant OxS in adult patients with mild to moderate atopic dermatitis (102,162).

**Figure 11:**
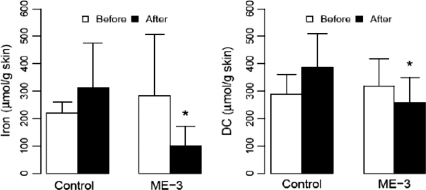
Content of iron and diene conjugates in the skin in patients with atopic dermatitis (AD), regularly (3 months) consuming probiotic strain ME-3. *p < 0.05 comparing the values before and after consumption.

The second DBRP pilot trial (162) was aimed at evaluation of the antioxidative effect of strain ME-3 on a seriously ill group of patients who had survived a stroke. The 21 patients (80.4±9.9 years), who had survived a brain stroke 12±6.6 days earlier, were randomly distributed into two groups. In addition to regular rehabilitation therapy that included ACE inhibitors, aspirin, diuretics and beta-blockers, the patients were assigned to consume for 3 weeks, twice a day, either capsules (3 ×per capsule 10^9^ cfu) of freeze-dried ME-3 (ME-3 group, 10 subjects) or placebo capsules (3 × 250 mg saccharose and microcellulose, control group, 11 subjects), respectively. The functional ability of the stroke patients was assessed before and after the 3-week treatment period using two clinical evaluation scales (Table VII). The Functional Independence Measure, FIM, assesses 18 activities of self-care, mobility, locomotion, communication and social cognition on a 7-point scale from fully independent to fully dependent. The Scandinavian Stroke Scale, SSS, is more specific to stroke patients and measures nine items: level of consciousness, mobility of eye, upper and lower limb, orientation, speech, facial tone and gait. The biochemical indices of sera were evaluated twice: before and after treatment (162). The baseline values of the ME-3 and control group were not statistically different. After rehabilitation and the course and consumption of the antioxidative probiotic, only the ME-3 group showed improved biochemical indices (GSSG, oxLDL, diene conjugates (DC), TAA) and the correlation between improved OxS-related indices and the functional ability scales (between SSS and oxLDL, *r* = −0.55, *p* < 0.05) and FIM (between FIM and GSSG/GSH, *r* = −0.63, *p* < 0.03). However, in the placebo group the FIM values got even better than in the ME-3 group, seemingly due to the lower starting level of the patients in the ME-3 group. In addition, the application of ME-3 capsules caused a significant decline in the values of inflammatory markers (hsCRP) not assessed in the control group.

**Table VII:** Clinical and biochemical evaluations of stroke patients: parameters at the baseline (before) and after an application period (after) by means of SSS and FIM scale and biochemical indices (mean±SED) (162).

Parameter	ME-3 group		Placebo group	
		
Clinical parameter	Before	After	Before	After
SSS	33 ± 13	42 ± 12^2^	37 ± 12	45 ± 9^1^
FIM	21 ± 19	40 ± 23^3^	32 ± 16	50 ± 16,^1^,^4^
Biochemical indices				
LDL-cholesterol (mmol/L)	3.9 ± 2.2	3.8 ± 1.9	3.2 ± 0.8	3.2 ± 1.1^4^
oxLDL (U/L)	121 ± 35	109 ± 35^1^	130 ± 23	128 ± 22^4^
DC (μmol/L)	50 ± 9	45 ± 8^3^	45 ± 16	45 ± 14
GSSG (μmol/L)	64 ± 16	52 ± 18^2^	73 ± 28	71 ± 18^4^
GSSG/GSH	0.07 ± 0.01	0.05 ± 0.01^1^	0.07 ± 0.02	0.06 ± 0.01
TAA (%)	34 ± 1	46 ± 3^2^	37 ± 1	35 ± 41^4^

DC, diene conjugates; FIM, Functional Independence Measure; GSSG, oxidized glutathione; GSSG/GSH, glutathione redox ratio; LDL, low-density lipoprotein; oxLDL, oxidized low-density lipoprotein; SSS, Scandinavian Stroke Scale; TAA, total antioxidant activity.

1 *p* < 0.05,

2 *p* < 0.01 and

3 *p* < 0.001 as compared to baseline value;

4 *p* < 0.05 as compared to the ‘After’ values in the ME-3 and placebo groups.

Figure 12 illustrates the effects of strain ME-3 for increasing the oxiresistance of LDL particles and lowering the level of oxLDL.

**Figure 12:**
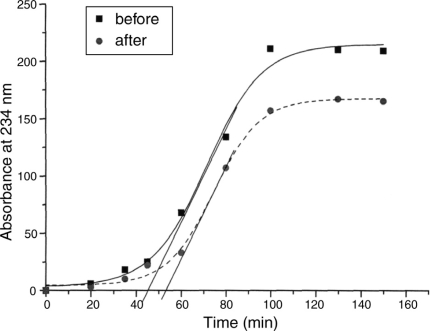
Increase of oxiresistance of low-density lipoprotein (LDL) particles (minutes) and lowering oxidized-LDL level (absorbance units) after using strain ME-3. Oxidation of LDL is measured on the basis of conjugated dienes at 234 nm.

Thus, this pilot trial shows the potential of strain ME-3 to be applied as an adjunct preparation to the ordinary rehabilitation therapy of patients in the course of recovering from a brain stroke.

## Possible mechanisms of effects of *L. fermentum* ME-3

In different experiments and volunteer and clinical trials, the administration of strain ME-3 has led to the improvement of the GI microbial ecology. More than a 10-fold increase of total lactobacilli counts in comparison with the individually different initial count was registered in the collected faecal samples, regardless of the probiotic formulation or daily dose applied. It was supposed that the metabolites secreted by strain ME-3 into the GI tract could be used as a substrate by other lactobacilli. The tolerance to different environmental factors and the successful passage of strain ME-3 through the GI tract has also been confirmed by culture and PCR-based methods (100,101).

At the same time, by adding *L. fermentum* strain ME-3 as a probiotic ingredient into a dairy product (yoghurt, cheese, milk), it was able to suppress the putative contaminants of food such as pathogenic *Salmonella* spp., *Shigella* spp., urinary tract infections caused by *E. coli, Staphylococcus* spp. The amounts of secreted SCFAs, the substantial amount of hydrogen peroxide (120,138,156) and production of NO by strain ME-3 were seemingly the main antimicrobial operators. The method for the simultaneous suppression of pathogens and the enhancement of the antioxidative activity of food was filed in a US patent application (171). Thus, the double functional properties of the probiotic strain *L. fermentum* ME-3 may protect the host against food-derived infections on one hand and help in the prevention of oxidative damage of food on the other hand. The intact cells and cell-free extract of strain ME-3 showed different potent antioxidative effects due to the expression of the antioxidant enzymes such as Mn-SOD and the components of the complete glutathione system (GSH, glutathione peroxidase and glutathione reductase). The antioxidative protection offered by strain ME-3 for the prevention of oxidative spoilage of semi-soft cheeses was assessed in an independent study in Finland (159).

Furthermore, we have looked for the implementation of the functional properties of strain ME-3 to improve antimicrobial defence and some metabolic functions in different hosts.

Experimental animal studies (161,164) have confirmed that the increase in total lactobacilli counts as much as the specific strain ME-3 antioxidative action in the gut eradicated live salmonellas and prevented the formation of typhoid nodules in experimental *Salmonella* Typhimurium infections, resembling typhoid fever in humans. For the first time it was shown that the antibiotic therapy of an invasive infection like enteric fever was more effective if administered together with a probiotic. However, it cannot be excluded that beside the antimicrobial and antioxidative effect of strain ME-3, immune enhancement by the probiotic also played a significant role.

Concerning the implementation of the antioxidative properties of strain ME-3 in humans, we have developed a method for enhancing the antioxidative activity of sera, i.e. administering strain ME-3 in a food product to humans comprising increases in TAA, TAS, and the lag phase of LDL and decreases in oxidized glutathione, oxLDL and BCD-LDL of sera (171). Different trials have shown that the application of strain ME-3 alleviates inflammation-and OxS-related shifts in gut, skin and blood (100–102,162). This is based on complicated cross-talk between strain ME-3 and host cells with the integrated influence of several factors such as the ability of strain ME-3 to apply a complete glutathione system, the expression of antioxidative enzymes in strain ME-3, the production of CLA and NO by strain ME-3, etc.

The question was raised as to how it was possible to exert a positive effect such as the reduction of OxS just by consuming a food product? Strain ME-3 of human origin was successful in surviving different fermentation processes of milk due to its good tolerance to low temperature, acid and salt (118,136), and was capable of the temporal colonization of the intestinal tract of the consumer.

The intestinal surface is an important host organism–environment boundary and the interactions of gut microbes inside the intestinal lumen and mucosal cells are important for the host, as was shown in the characterization of the composition and metabolic activities of intestinal microbiota in the first sections of the review. An impaired environment such as the imbalance of GI microbiota, but also the increase of lipid peroxidation and decrease of the reduced GSH both at the intestinal surface and in the intestinal cells, are the mighty modulators causing different unhealthy outcomes in the host. That these modulators of the intestinal mucosal status can be repaired by the administration of strain ME-3 was directly confirmed in a mouse model of experimental S. Typhimurium infection (161,164). In this process the involvement of the glutathione system is crucial as GSH, besides its role as a crucial antioxidant, is the principal redox controller for a number of processes in cells. Glutathione-related information has impact for strain ME-3 regarding at least two aspects: a) a recent adapted conception of OxS is advanced as ‘a disruption of redox signalling and control’ (145,172) that emphasizes an impact of GSH and its redox ratio as good tools for the quantification of OxS and the signalling role of GSH, described also by us (173,174); and b) there exists the possibility for the effective participation of strain ME-3 in both enzymatic and non-enzymatic glutathione-driven protection. Besides, the data that just *L. fermentum* as a species significantly counteracted the depletion of colonic glutathione content induced by some inflammatory processes (175) also supported our understanding. In addition, there exists a correlation between glutathione redox ratio and DNA oxidative damages (176).

Further, our research has shown that the improvement of the intestinal extra- and intracellular environment yielded beneficial changes of some general/systemic biochemical indices of the host. This was proven by the administration of strain ME-3 to healthy volunteers and atopic adults leading to a reduction of lipid peroxidation and a counterbalance of the glutathione system both in blood and in skin. Moreover, in several conducted trials we have seen the positive effect of strain ME-3 on the blood LDL fraction: the prolongation of its resistance to oxidation, the lowering of the content of oxLDL (potent inflammatory and atherogenic factor) and BDC-LDL and the enhancement of the total antioxidative capacity of sera (100,101,167).

In our recent investigation of elderly persons over 65 years, the lower content of oxLDL was significantly predicted by the higher count of live lacto-bacilli in the GI tract (M. Mikelsaar et al., unpublished observations). Seemingly, both the particular antioxidative characteristics of strain ME-3 and the increase in lactobacilli counts induced by its administration could be responsible for the registered impact on the host lipid metabolism.

The status of OxS and blood lipoprotein are both related to the development of different diseases, including inflammation-related diseases and CVD (see above). Recently in *Circulation* (177) the pathophysiological continuum that traditional cardiovascular risk factors all promote OxS and endothelial dysfunction – the first steps in a cascade of pathological events – was highlighted. OxS leads to the overproduction of oxLDL and the latter has a great impact on the development of atherosclerosis. For example, the higher levels of circulating oxLDL are strongly (much more than LDL-cholesterol) associated with an increased incidence of metabolic syndrome already in people who are currently young and healthy according to a large population-based study (178). It was previously shown that oxLDL is an important determinant of structural changes of the arteries already in asymptomatic persons (147, 179). Recent data gathered from the literature suggest that the increased production of atherogenic and inflammatory oxLDL within the vessel wall suppresses several immunity-related cells, including regulatory T cells (180) exerting antiatherogenic and antiallergic effects. In addition, it is widely accepted that post-prandial abnormal events are crucial as regards the development of CVD (181).

The systemic influence of strain ME-3 on host OxS indices has also been assessed by the decline of the values of isoprostanes and 8-OHdG in urine (100,101,162). Both indices are accepted as very informative markers for human systemic OxS burden (144,145). Evidently the systemic antioxidative effect of strain ME-3 starts from the alleviation of the OxS- and inflammation-related abnormalities in the intestinal cells that lead to the assembling of particles of chylomicrons, LDL and HDL with a higher bioquality with lower levels of harmful oxidation products and higher concentrations of antioxidant enzymes in their particles. Furthermore, the increased bioquality of assembled lipoprotein particles is bound to aid the improvement of their metabolism/circulation in the host body. This is one of the possible explanations why strain ME-3 exerted the prolonged resistance of the blood lipoprotein fraction to oxidation, lowered the level of oxLDL and enhanced the total antioxidative capacity of sera in both healthy and diseased strain ME-3 consumers (100,101,162,163). Our recent data have shown that administration of strain ME-3 alleviates the post-prandial elevation of triglyceride levels in the blood, and improves HDL bioquality (elevation of antioxidative enzyme level in HDL particles) (162,163). This new HDL-related antioxidative information is supported both by our findings of anti-inflammatory effects of strain ME-3 on the liver (164) and by a hepato-protective role for paraoxonase against inflammation, fibrosis and liver disease mediated by OxS (182).

The necessity for new approaches in global cardiovascular risk reduction has become widely accepted (183). In the prevention of cardiovascular risk the anti-inflammatory agents and antioxidants are considered as a possible ‘third great wave’ (184). The prevention complexes of several diseases could become more successful by also including probiotics with multivalent biopotency. Hopefully the antimicrobial and antioxidative probiotic *L. fermentum* ME-3 has earned its place in this putative list of health promoters.

## Summary and conclusions

The probiotics aimed at stabilizing the microbial communities and the health-promoting effects have an important position in the medical health care of different age groups and diseased persons. This paper describes the process of the discovery, identification and molecular typing of the strain of human origin, *Lactobacillus fermentum* ME-3 (DSM-14241), elaborated according to the regulations of WHO/FAO (2002). In this review the authors have attempted to compile the available information concerning the ability of *L. fermentum* ME-3 to protect the host from different diseases induced by pathogenic bacteria (*Salmonella* spp., *Shigella* spp. and urinary tract infections cause by *E. coli*), inflammation and oxidative stress.

Safety and health-promoting studies of the probiotic strain cited in this report were carried out by assessing a large number of microbiological, biochemical and clinical indices. This strain is still unique among *Lactobacillus* species, having both antimicrobial and physiologically effective antioxidative properties. Tartu University has patented this probiotic strain in Estonia (priority June 2001, patent in 2006), Europe (pending), Russia (patent in 2006) and USA (patent in 2007).

The functional efficacy of this multipotential probiotic has been proven by the eradication of pathogenic microbes and the reduction of liver and spleen granulomas in *Salmonella* Typhimurium-infected mice treated by the combination of ofloxacin and *L. fermentum* strain ME-3. When used in capsules or foodstuffs (yoghurt, kefir, cheese) *L. fermentum* ME-3 expresses several health-promoting effects. It has been shown in different subjects (healthy volunteers, patients) with different clinical study designs (including double-blind, placebo-controlled, crossover studies) that this probiotic increased the counts of lactobacilli in the intestinal tract, lowered the 8-isoprostanes content in urine, increased the antioxidative activity, lowered the content of atherogenic oxLDL, and improved post-prandial lipid as well as oxidative stress status in sera, thus demonstrating an anti-atherogenic effect.

The elaboration of the probiotic *L. fermentum* strain ME-3 has drawn on wide international cooperative research and has taken more than 12 years altogether. The new probiotic products containing strain ME-3 have been successfully marketed and sold in Baltic countries and Finland.
